# Supplementary material for: MetaRibo-Seq measures translation in microbiomes
Source: Nat Commun. 2020 Jun 29;11:3268. doi: 10.1038/s41467-020-17081-z (PMC7324362; doi:10.1038/s41467-020-17081-z)
Supplement: Supplementary file 10 — Supplementary Data 7 [file 41467_2020_17081_MOESM10_ESM.zip › File2/Confidence_High_Taxonomy/328201_out.krona.html]

Javascript must be enabled to view this page.

members
magnitude
magnitudeUnassigned
count
unassigned
taxon
rank

328201\_out

6

superkingdom
6
2

6
phylum
1239

186801
class
6

6
order
186802

6
family
541000

216851
6
genus

853
6
species

SRS011134\_contig\_number\_contig-100\_462.311995SRS047044\_contig\_number\_168SRS076929\_contig\_number\_7067SRS076976\_contig\_number\_9083SRS143895\_contig\_number\_5640SRS144506\_contig\_number\_18432
